# The research progress and prospects of circadian rhythm in obesity: a bibliometric analysis

**DOI:** 10.3389/fnut.2024.1499984

**Published:** 2025-01-07

**Authors:** Ye Dou, Xiaojin Guo, Xuefei Wang, Aolong He, Fanghe Li, Kuo Gao

**Affiliations:** ^1^School of Traditional Chinese Medicine, Beijing University of Chinese Medicine, Beijing, China; ^2^Beijing Tongzhou District Hospital of Integrated Traditional Chinese and Western Medicine, Beijing, China

**Keywords:** circadian rhythm, obesity, bibliometric analysis, visualization, CiteSpace, VOSviewer

## Abstract

**Background:**

Numerous studies have shown a link between circadian rhythms disruptions and a higher risk of obesity. This article aims to conduct an extensive bibliometric analysis to deepen our understanding of the relationship between circadian rhythms and obesity.

**Methods:**

The literature related to the circadian rhythm of obesity, published from the inception of the Web of Science Core Collection (WoSCC) until June 30, 2024, was extracted from the WoSCC databases (SCIE, SSCI, ESCI). Using CiteSpace, Vosviewer, WPS, and other software, this paper examines the publication trends, including the number of papers, countries/regions, institutions, authors, journals, references, and keywords.

**Results:**

A total of 2,870 articles were included in this analysis, revealing a consistent year by year increase in research on the circadian rhythm of obesity. These publications originate from 460 institutions in 88 countries. Among the authors analysis, Garaulet, Marta was the most prolific, and Turek FW was the most co-cited. Proceedings of the National Academy of Sciences of the United States of America emerged as the journal with the highest number of publications, and American Journal of Physiology had the highest centrality. The most frequently used keywords were “obesity,” “circadian rhythm,” “circadian clock,” “metabolic syndrome,” “metabolism.” Additionally, research areas involving intermittent fasting, restricted feeding, and gut microbiota were rapidly developing and represented the forefront of research on circadian rhythms and obesity.

**Conclusion:**

Our study demonstrates that research on circadian rhythms in obesity has been rapidly expanding, with increasingly in-depth exploration of the topic. It is recommended to strengthen cooperation between countries and institutions to jointly promote research in this field. The gene expression of obesity is an early hotspot in the study of circadian rhythm and obesity, and emerging research areas such as intermittent fasting, restricted feeding, endothelial nitric oxide synthase and gut microbiota will become significant hotspots and trends in the field of circadian rhythm and obesity. These findings provide researchers critical directions for future studies and may have significant implications for clinical practice and public health policy.

## Graphical abstract

Frontiers and Hotspots in Circadian Rhythm and Obesity Research: A Bibliometric Analysis. This study utilized the Web of Science, CiteSpace, and VOSviewer to analyze research on obesity and circadian rhythms. The results cover publication trends, key countries, institutions, references, authors, journals, and keywords. Emerging fields include circadian gene expression, intermittent fasting, time-restricted feeding, endothelial nitric oxide synthase, and gut microbiota, with therapeutic targets in circadian rhythms offering promising prospects for obesity treatment.

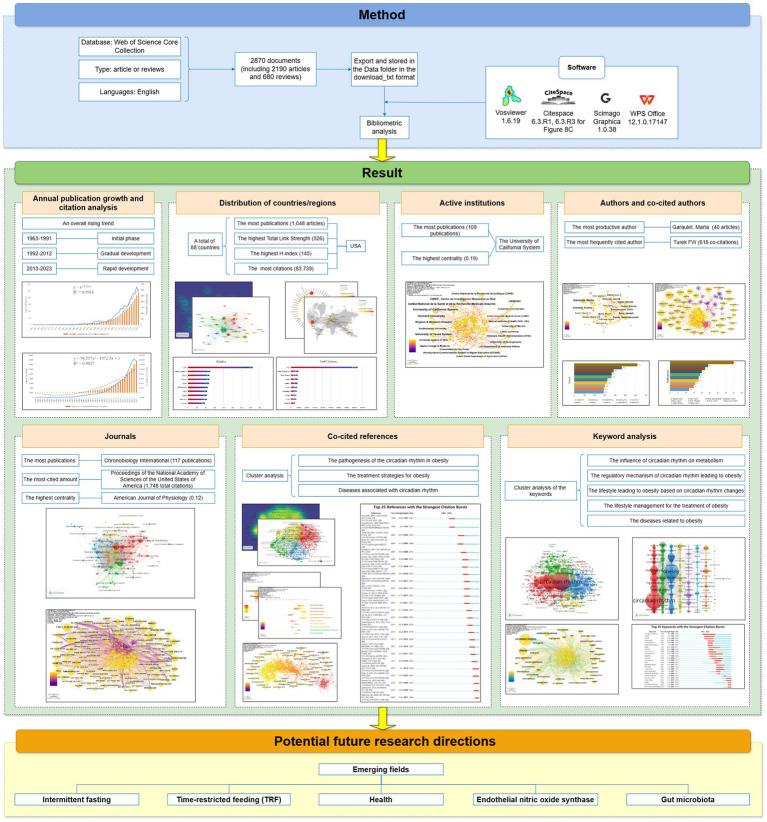

## 1 Introduction

Obesity is a common chronic disease characterized by the excessive accumulation or abnormal distribution of fat, which poses significant health risks and requires ongoing management (1). According to the World Health Organization, adults are considered overweight if they have a body mass index (BMI) of 25 kg/m^2^ or higher, and as obese if their BMI is 30 kg/m^2^ or more. For children, age must be taken into account when defining overweight and obesity (2). The pathogenesis of obesity is complex, involving biological, psychosocial (3, 4), genetic (5), and socio-economic (1) factors, as well as diverse pathways and mechanisms that contribute to adverse health outcomes (6). Obesity is closely linked to an increased risk of several chronic diseases, including cardiovascular disease (6), type 2 diabetes (7), various cancers (8), osteoarthritis (9) and several other diseases, and it poses serious public health challenges. Over the past decades, the global obesity pandemic has intensified significantly. From 1990 to 2022, the prevalence of obesity more than doubled worldwide. In 2022, 2.5 billion adults aged 18 and older were classified as overweight, including over 890 million who were obese (2). Given these alarming trends, obesity continues to be a major global public health challenge that requires urgent attention and intervention.

The term “circadian” refers to processes that follow a roughly 24 h cycle (10). Circadian rhythms are endogenous regulators in cells or organisms that coordinate physiological and behavioral activities, playing a vital role in numerous physiological processes (11). These rhythms are controlled by the central clock, located in the suprachiasmatic nucleus (SCN) of the hypothalamus, as well as the peripheral clocks throughout the whole body (12). Extensive studies have provided insights into the mechanism by which circadian rhythms contribute to obesity, involving the interaction of various physiological, environmental, and genetic factors (13). The circadian rhythm system regulates various metabolic processes related to obesity, including glucose and cholesterol metabolism (14, 15). In glucose metabolism, sleep deprivation reduces brain glucose utilization, resulting in increased glucose concentrations in peripheral tissues. This elevation may promote the development of insulin resistance and lead to obesity (16). Researchers have found that day and night reverse feeding aggravates the rise in blood glucose and lipid levels caused by a high-fat diet in 8–10-week-old mice. Notably, daytime feeding results in a more obvious increase in weight gain, fasting blood glucose concentration, and lipid concentration (17). Similarly, the plasma levels of hormones involved in metabolic processes, such as glucagon and leptin, also follow circadian rhythms (18, 19). Glucagon-like peptide-1 (GLP-1) and glucose-dependent insulinotropic polypeptide (GIP) enhance insulin secretion and inhibit food intake to control glucose homeostasis. Under similar dietary conditions, plasma concentrations of glucagon, GLP-1 and GIP were found to be higher in the daytime than at night, while the levels of GLP-1 and GIP were lower at night, which may be the reason for the frequent increase of food intake at night, thereby increasing the risk of obesity (18). Leptin, an essential pleiotropic adipokine, plays a key role in regulating energy balance (20). A study showed participants with short sleep duration reduced leptin levels, increased auxin-releasing peptides, and heightened hunger and appetite, all of which can lead to obesity (21, 22). Additionally, the environment and the resulting circadian rhythm disorders are also closely related to obesity. An unnatural distribution of light and dark within 24 h can interfere with the early development of the nervous system of mice and change the weight regulation during the early growth. The reintroduction of fragmented day-night cycles in adulthood has also been led to overeating in adulthood (23). Research on the role of circadian rhythm genes in obesity has begun very early and continues to develop. As early as 2005, Turek FW found that Clock mutant mice fed a high-fat diet became obese at a young age and exhibited several metabolic and endocrine abnormalities consistent with metabolic syndrome such as increased appetite, obesity, hyperleptinemia, hyperlipidemia, hyperglycemia (24). Since then, research on the relationship between obesity and circadian rhythms has continued to advance. A recent study revealed, for the first time, the correlation between the methylation of circadian rhythm genes BMAL1 and Clock and various obesity-related indices, including body mass index, waist circumference, fasting blood glucose, total cholesterol, low density lipoprotein cholesterol, high-density lipoprotein cholesterol, triglycerides, HOMA-IR, leptin, adiponectin and resistin. This study opens up possibilities for exploring the epigenetic mechanisms by which circadian rhythm regulation influences obesity (25).

The systematic review is a structured process of collecting, screening, evaluating, and analyzing all relevant literature on a specific question or research topic. It encompasses both qualitative and quantitative reviews, aiming to provide an unbiased synthesis of studies, identify contradictions, gaps, and inconsistencies in the evidence, and explore their underlying causes. Ultimately, it seeks to propose new theories or critically assess existing ones. Different from systematic reviews, bibliometrics is the study of academic publishing that uses mathematical and statistical methods to analyze publishing trends and highlight relationships between published works (26). Based on the complexity and quantity of available information, this approach can intuitively visualize countries, institutions, authors, journals, literatures and keywords, allowing researchers to quickly gain a deep understanding of the thematic evolution, research hotspots, and emerging trends within specific fields (27). However, no bibliometric study has been conducted on the relationship between obesity and circadian rhythm. Thus, in this study, we employed bibliometric methodologies to assess the research status and current focus of studies on circadian rhythm in obesity, and to explore potential directions for future research.

## 2 Materials and methods

### 2.1 Data source and search strategy

Using the WoSCC (SCIE/SSCI/ESCI), a comprehensive literature search was conducted without a start date set and continued until June 30, 2024. The Web of Science core collection was selected for bibliometric analysis because it contains a substantial number of biomedical studies (28), tracks older citations more effectively (29), and classifies journals more accurately than Scopus (30). The study used a search formula of “(TS = (“Circadian Rhythm*” OR “Twenty* Four Hour Rhythm*” OR “Nyctohemeral Rhythm*” OR “Nycthemeral Rhythm*” OR “Diurnal Rhythm*” OR “Circadian Clock*” OR “Clock System*”) AND TS = (“Obesity” OR “Obese” OR “Obesities” OR “Overweight” OR “Over weight” OR “Adiposity”)).” A total of 3,026 records were retrieved, with only two types of documents—articles and reviews— included in the current research. The literature was limited to the English-language publications. Finally, 2,870 records were utilized in this study. To ensure the accuracy of data updates, all of the above processes were completed within a 24 h period on July 19, 2024.

### 2.2 Data analysis tool and statistical methods

In this study, WPS Office (version 12.1.0.17147), CiteSpace (version 6.3.R1, 6.3.R3 for Figure 8C), and VOSviewer (version 1.6.19) were utilized to conduct the bibliometric analysis. CiteSpace is a JAVA-based software designed for visual exploration and information discovery in bibliographic databases (31). By utilizing a progressive knowledge domain visualization approach, CiteSpace identifies and visualizes trends and patterns in scientific literature. It can visually map highly cited and influential articles, areas of specialty within a knowledge domain, and the emergence of new research subjects (32). The parameters for the analysis were set as follows: time span (January 1963–June 2024), years per slice (1), node type (select one at a time, such as keyword, reference, or cited author), term source (title, abstract, author keywords, keywords plus), selection criteria (threshold: c, cc, and ccv depending on particular situations), pruning (pathfinder, pruning sliced networks), and visualization (default parameters).

VOSviewer, a freely available program, is widely used for document analysis (33). It excels in co-occurrence and co-citation analysis, offering the ability to present expansive bibliometric maps in a visually simple and understandable format (33, 34). The parameter settings of VOSviewer in this study were as follows: counting method (full counting), Type of Analysis (co-occurrence), Unit of Analysis (All Keywords), Minimum number of occurrences of a keyword (5), Number of keywords to be selected (1000).

**Figure 1:**
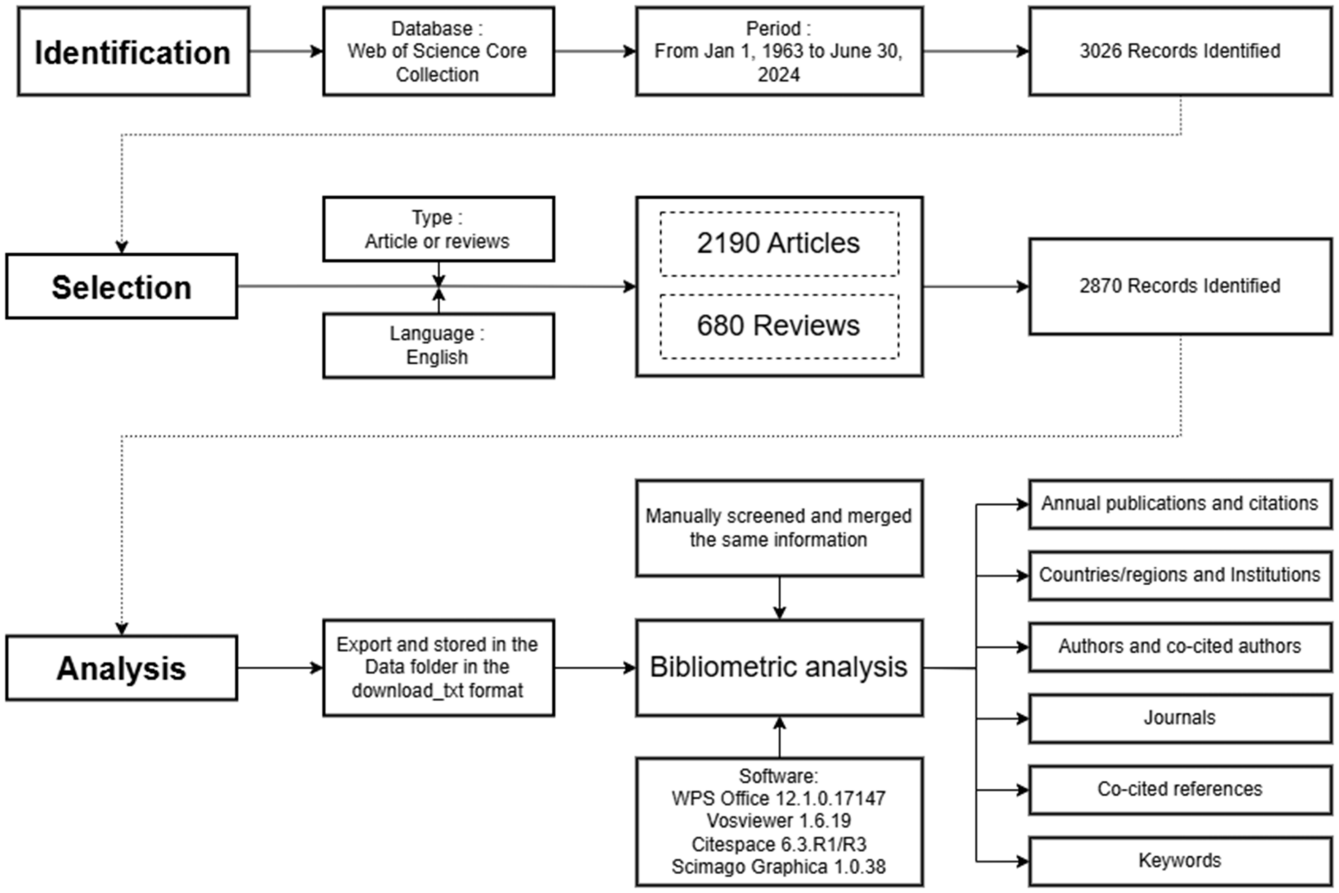
Search strategy and data analysis methods flow chart.

Due to variations in the presentation of specific authors, institutions, or keywords, we manually filtered and merged the same information prior to formal analysis. Figure 1 shows the detailed search strategy and data analysis methods.

## 3 Results

### 3.1 Annual publication growth and citation analysis

**Figure 2:**
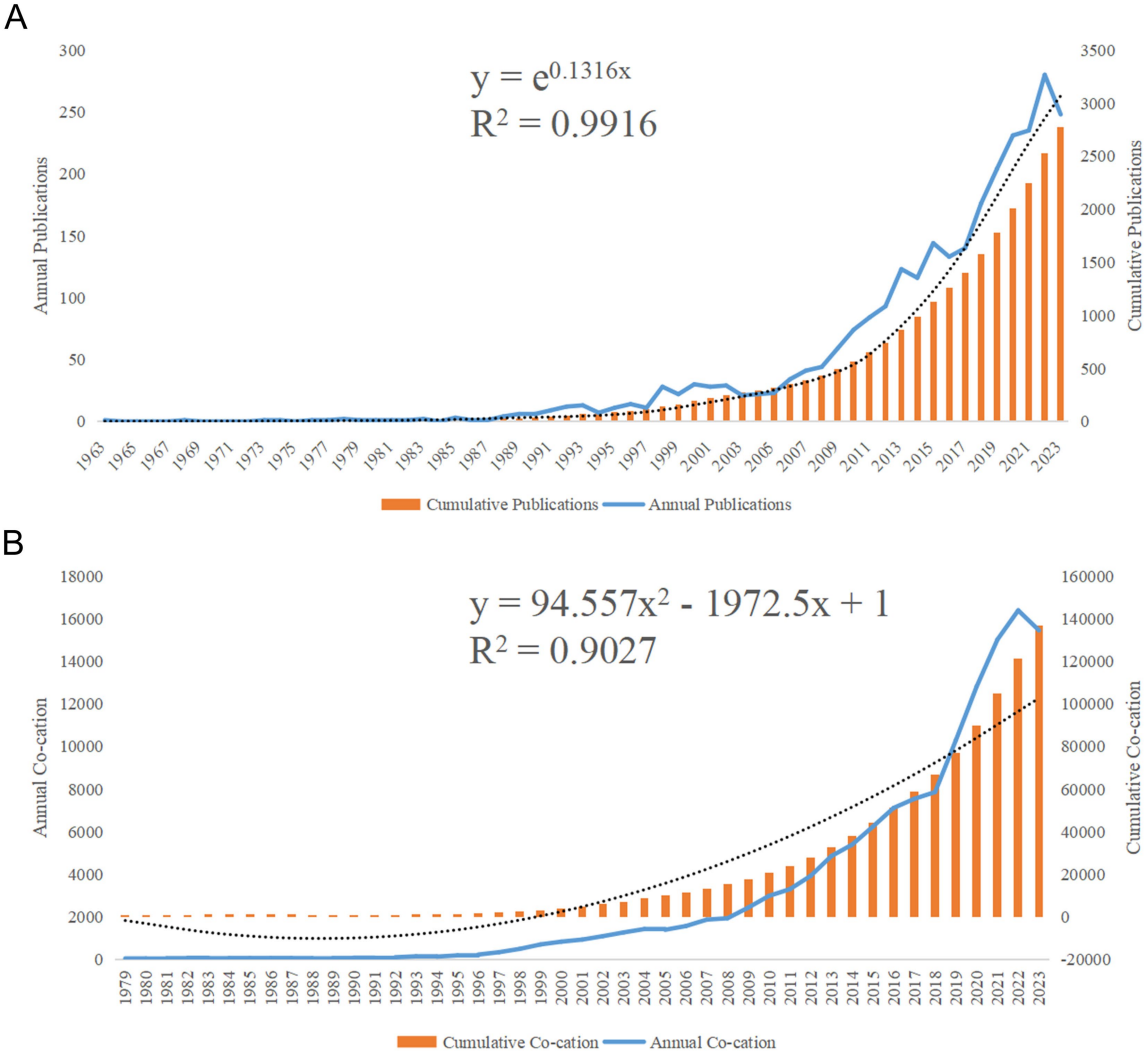
Articles related to the study of the circadian rhythm in obesity. **(A)** The annual and cumulative number of publications from 1963 to 2023. **(B)** Annual and cumulative number of citations (Nc) from 1979 to 2023.

A total of 2,870 relevant papers were obtained, accumulating 125,783 total nonself-cited citations, with an average of 50.21 citations per article. The H-index of all articles was 171. The annual number of publications (Np) and annual citations (Nc) related to the circadian rhythm research in the context of obesity are shown in Figures 2A,B. Since only literature from the first half of 2024 was available, the data from 2024 were not included in Figures 2A,B. As shown in Figure 2, although some fluctuations in the past 60 years, the overall trend shows a rise in both publications and citations. Figure 2B shows a steady increase in the number of annual citations. Due to the low annual citation in the years prior to 1979, Figure 2B records starting from 1979. In Figure 2A, the first study on this topic was published in 1963. Based on the annual research output, the growth in the number of papers on circadian rhythm and obesity can divided into roughly three stages. The first stage, from 1963 to 1991, represents the initial phase, with relatively little research produced before 1991. The second stage, spanning 1992 to 2012, was marked by gradual development, as evidenced by the steady rise in annual output, indicating growing attention to this field. The third stage was the rapid development from 2013 to 2023. During this period, an average of over 100 articles were published annually, peaking at 280 in 2022, with an average annual citation count of 16,403. This trend highlights the increasing focus of academics on the circadian rhythm in obesity research.

### 3.2 Distribution of countries/regions

**Figure 3:**
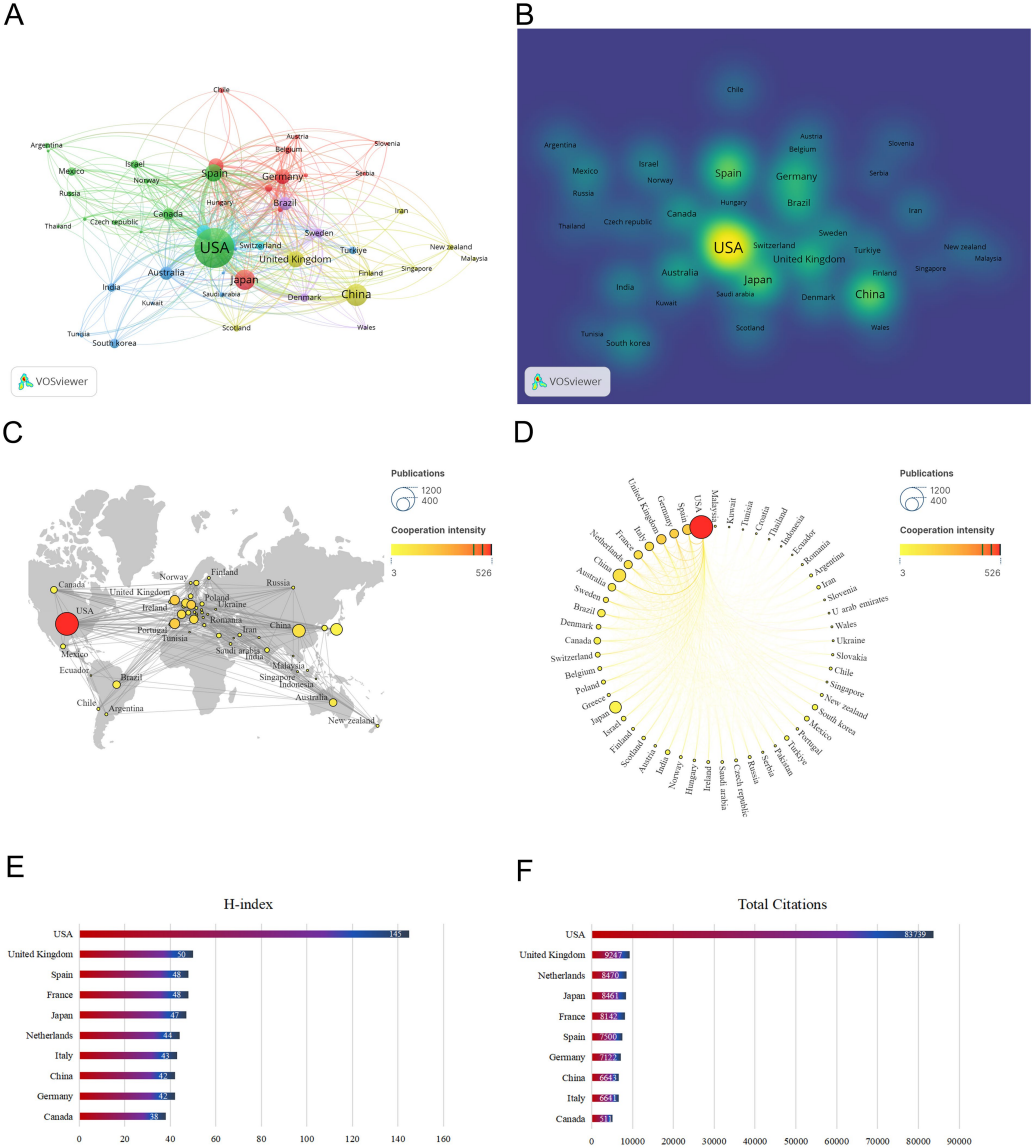
Leading countries related to circadian rhythms in obesity research. **(A)** Map of country collaborations. The circle in the graph represents the country, and the connection between the circles represents the cooperation between the countries. **(B)** Density map of countries. **(C)** The world map of the intensity of cooperation between countries. **(D)** A circle diagram that evaluates international collaboration among clusters. **(E)** The top 10 countries in H-index. **(F)** The co-citation frequency ranked the top 10.

A total of 88 countries have published articles on obesity and circadian rhythm to date. The minimum documents quantity of a country was set at 5, and 53 of these 88 countries met the thresholds. As shown in Figures 3A,B and Table 1, the USA leads with the most publications (1,048 articles), significantly surpassing the second-ranked and third-ranked countries, China (322) and Japan (275), respectively. Figures 3C,D display a map of international cooperation by country or region. The USA also exhibits the highest Total Link Strength (526), demonstrating the strongest international cooperation, followed by Spain (201) and Germany (197). The number of publications is represented by the size of each node. The color of the nodes and the thickness of the lines indicate the strength of collaboration between countries. The data show that the significantly closer cooperation between the United States and countries such as China, Spain, and Germany. One method of evaluating researchers’ academic contributions and predicting future achievement is the H-index (35, 36). As shown in Figure 3E, the USA has the highest H-index (145), followed by the UK (50) and Spain (48). In terms of the total citations (Figure 3F), the USA leads with the 83,739 citations, followed by the United Kingdom with 9,247 citations, and the Netherlands with 8,470 citations. Results show that Europe, Asia and the USA are the primary regions contributing to publications in this field, with the USA holding a dominant position in this field of research on obesity and circadian rhythm.

**Table 1:** The top 10 countries in the number of publications.

Rank	Country	Publications	Total citations	Average citation	H-index	Total link strength
1	USA	1,048	83,739	79.9036	145	526
2	China	322	6,643	20.6304	42	123
3	Japan	275	8,461	30.7673	47	40
4	Spain	196	7,500	38.2653	48	201
5	United Kingdom	177	9,247	52.2429	50	194
6	Germany	152	7,122	46.8553	42	197
7	Italy	145	6,641	45.8	43	145
8	France	140	8,142	58.1571	48	133
9	Netherlands	135	8,470	62.7407	44	125
10	Australia	117	4,080	34.8718	34	105

### 3.3 Authors and co-cited authors

#### 3.3.1 Authors

**Figure 4:**
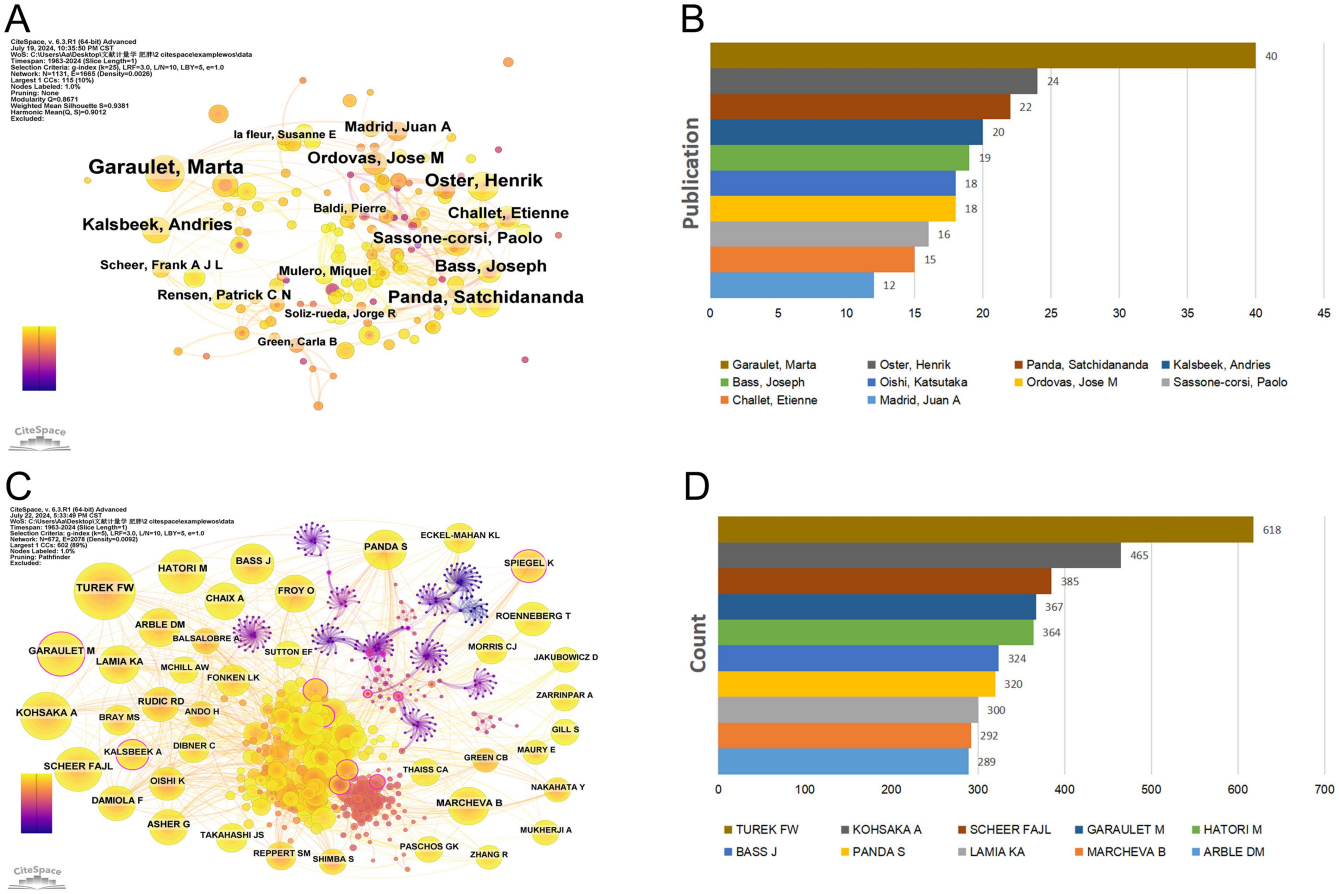
Authors involved in the study of the circadian rhythm in obesity. **(A)** Author co-occurrence visualization based on CiteSpace. **(B)** The top ten authors in the number of publications. **(C)** Co-citation author network diagram based on CiteSpace. **(D)** Top 10 co-citation authors.

A visual analysis using CiteSpace was conducted to map the contributions of these authors, since the publication of the first article on circadian rhythm in obesity in 1963. In this map, the size of each node represents the author’s total number of publications. The width of the connection line indicates the degree of cooperation between authors. A larger node means more publications. The map of authors (Figure 4A) consists of 1,131 nodes and 1,665 edges, with network density of 0.0026. The top 10 authors contributed 204 articles, accounting for 7.11% of the total publications (Table 2; Figure 4B). The most productive author is Garaulet, Marta (40 articles, 1.39%), followed by Oster, Henrik (24 articles, 0.84%), Panda, Satchidananda (22 articles, 0.77%), Kalsbeek, Andries (20 articles, 0.70%) and Bass, Joseph (19 articles, 0.66%).

**Table 2:** The top 10 authors in the number of publications or co-citation.

Rank	Author	Institutions	Publications	Rank	Author	Institutions	Count
1	Garaulet, Marta	University of Murcia	40	1	TUREK FW	Northwestern University	618
2	Oster, Henrik	University of Lubeck	24	2	KOHSAKA A	Wakayama Medical University	462
3	Panda, Satchidananda	Salk Institute	22	3	SCHEER FAJL	Harvard Medical School	385
4	Kalsbeek, Andries	University of Amsterdam	20	4	GARAULET M	University of Murcia	367
5	Bass, Joseph	Northwestern University	19	5	HATORI M	Jichi Medical University	364
6	Ordovas, Jose M	Tufts University	18	6	PANDA S	Salk Institute	320
7	Oishi, Katsutaka	University of Tsukuba	18	7	BASS J	New York Genome Ctr	319
8	Sassone-corsi, Paolo	University of California Irvine	16	8	LAMIA KA	Scripps Research Institute	300
9	Challet, Etienne	Universite de Strasbourg	15	9	MARCHEVA B	Northwestern University	292
10	Madrid, Juan A	University of Murcia	12	10	ARBLE DM	Marquette University	289

#### 3.3.2 Co-cited authors

Figure 4C presents a visual analysis of the network of co-cited authors, while Table 2 and Figure 4D highlight the top 10 authors most frequently cited in references. With 618 co-citations, Turek FW tops the list with 618 co-citations, followed by KOHSAKA A with 462 co-citations, SCHEER FAJL with 385, GARAULET M with 367, and HATORI M with 364 co-citations. In terms of centrality, BRAY GA (0.37), VANCAUTER E (0.36), and MOORE RY (0.28) stand out, suggesting their roles as key “bridge” authors in the field.

### 3.4 Active institutions

**Figure 5:**
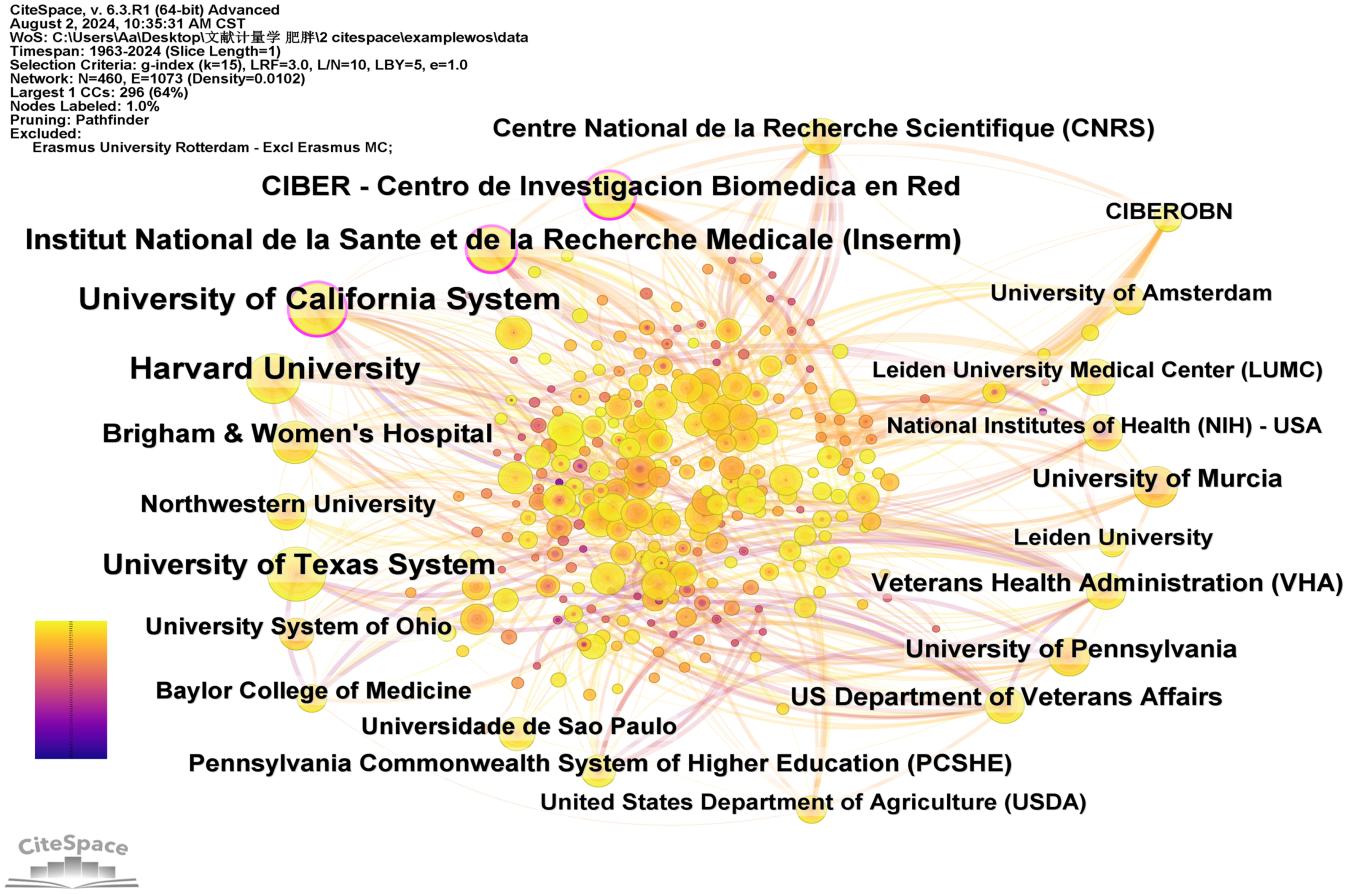
Network of institutions carrying out research on the circadian rhythm of obesity.

Table 3 lists the top 12 institutions ranked by the centrality in circadian rhythm and obesity research, as well as the top 11 institutions ranked by publication quantity. The University of California System leads with 109 publications, followed by Harvard University with 103 publications, Institut National de la Sante et de la Recherche Medicale (Inserm) with 73 publications, the University of Texas System also with 73 publications, and Centro de Investigacion Biomedica en Red (CIBER) with 59 publications. Figure 5 shows the network cooperation map among various institutions. In this figure, each node represents an institution, with larger nodes indicating a higher publication output. Links connecting nodes depict institutional cooperation, with the color of the links indicating the start time of collaboration and the thickness of the lines representing the strength of collaboration. Institutions with high centrality (≥0.10), represented by a purple circle around the node, are positioned as potential leaders of significant breakthroughs and serve as bridges within the network (37). The University of California System (0.19), Inserm (0.16), and CIBER (0.10) rank first through third in centrality, respectively. Overall, the figure demonstrates relatively close cooperation among these leading institutions in the field.

**Table 3:** The top 10 institutions with the largest number of publications and the top 10 institutions with centrality.

Rank	Institutions	Count	Rank	Institutions	Centrality
1	University of California System	109	1	University of California System	0.19
2	Harvard University	103	2	Institut National de la Sante et de la Recherche Medicale (Inserm)	0.16
3	Institut National de la Sante et de la Recherche Medicale (Inserm)	73	3	CIBER – Centro de Investigacion Biomedica en Red	0.10
4	University of Texas System	73	4	Harvard University	0.08
5	CIBER – Centro de Investigacion Biomedica en Red	59	5	Brigham & Women’s Hospital	0.07
6	Brigham & Women’s Hospital	52	6	US Department of Veterans Affairs	0.07
7	Centre National de la Recherche Scientifique (CNRS)	42	7	Pennsylvania Commonwealth System of Higher Education (PCSHE)	0.07
8	US Department of Veterans Affairs	41	8	CIBEROBN	0.06
9	University of Murcia	41	9	University of Texas System	0.05
10	Veterans Health Administration (VHA)	40	10	Veterans Health Administration (VHA)	0.05
11	University of Pennsylvania	40	11	University of Pennsylvania	0.05
			12	Helmholtz Association	0.05

### 3.5 Journals

**Figure 6:**
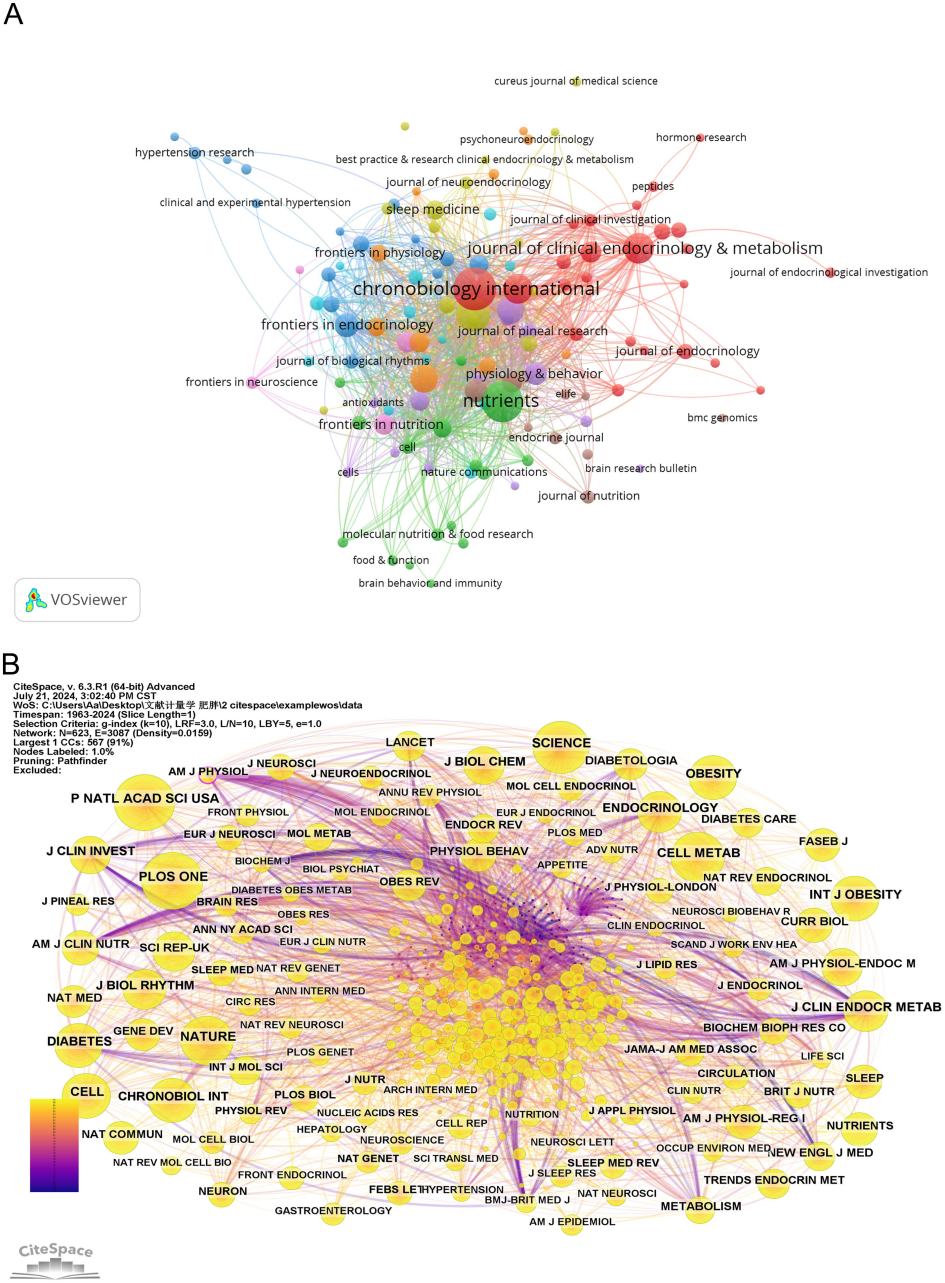
Visualization of journals of the circadian rhythm in obesity. **(A)** Visual analysis of source journals based on VOSviewer. **(B)** Citation journal visualization based on CiteSpace.

Based on the full-count bibliographic coupling analysis of journals, most publications on circadian rhythm and obesity were released in 119 academic journals (Figure 6A). The journal with the highest number of publications on this topic is Chronobiology International (117 publications), followed by Nutrients (109 publications) and Plos One (76 publications). According to a source analysis of the included literature in Figure 6A, the top 10 journals with the most published papers are situated in the JCR partition of Q1 or Q2, indicating that these journals are of reliable quality and should be prioritized by researchers seeking to publish their work. In terms of co-citation, the most-cited journal is PROCEEDINGS OF THE NATIONAL ACADEMY OF SCIENCES OF THE UNITED STATES OF AMERICA (1,748 total citations), followed by SCIENCE (1,607 total citations), and NATURE (1,490 total citations), as shown in Figure 6B. Table 4 shows that AMERICAN JOURNAL OF PHYSIOLOGY (0.12) has the highest centrality, followed by the AMERICAN JOURNAL OF CLINICAL NUTRITION (0.09) and LANCET (0.09). It indicates that these journals exert a substantial influence on the field of circadian rhythm and obesity research.

**Table 4:** Top 10 journals in terms of number of co-citation or centrality.

Rank	Journal	Count	JCR partitions	Rank	Journal	Centrality	JCR partitions
1	Proceedings of the National Academy of Sciences of the United States of America	1748	Q1	1	American Journal of Physiology	0.12	Q1
2	Science	1,607	Q1	2	American Journal of Clinical Nutrition	0.09	Q1
3	Nature	1,490	Q1	3	Lancet	0.09	Q1
4	Plos One	1,462	Q1	4	Nature	0.08	Q1
5	Cell Metabolism	1,279	Q1	5	Biochemical Journal	0.08	Q2
6	International Journal of Obesity	1,250	Q1	6	Bmj-British Medical Journal	0.07	Q1
7	Diabetes	1,228	Q1	7	Science	0.06	Q1
8	Cell	1,196	Q1	8	Journal of Clinical Endocrinology & Metabolism	0.06	Q1
9	Journal of Clinical Endocrinology & Metabolism	1,176	Q1	9	Journal of Biological Chemistry	0.06	Q2
10	Endocrinology	1,145	Q2	10	Endocrinology	0.05	Q2
				11	Chronobiology International	0.05	Q2
				12	Life Sciences	0.05	Q1
				13	Bmj-British Medical Journal	0.05	Q1

### 3.6 Co-cited references

**Figure 7:**
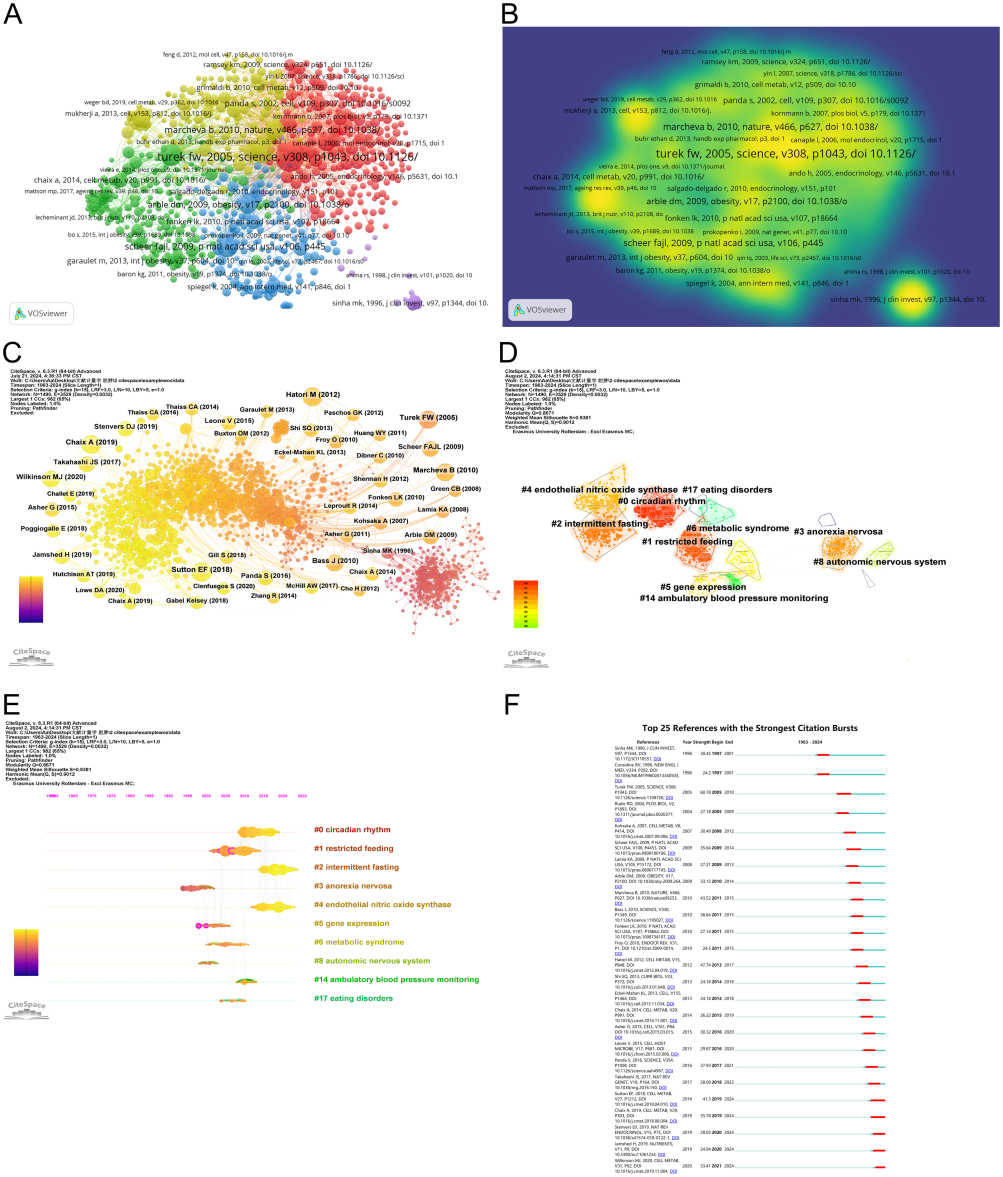
Visualization of co-cited literature on the circadian rhythm research in obesity. **(A)** References co-citation network based on VOSviewer. **(B)** The density visualization of co-cited references based on VOSviewer. **(C)** References co-citation network based on CiteSpace. **(D)** Cluster analysis of co-cited references based on CiteSpace. **(E)** A timeline of the 10 largest clusters. **(F)** Top 25 references with the strongest citation bursts.

Co-citation references refer to the literature that is cited by multiple papers simultaneously, indicating the degree of interconnectedness between those references. VOSviewer identified the top 5 references with the highest number of co-citations (Figures 7A,B; Table 5). Among them, the most-cited article was published by Turek, FW, who demonstrated through a controlled trial involving wild-type (WT) mice and mice homozygous for the Clock mutation. The circadian clock gene network plays a crucial role in the energy balance of mammals, which is associated with many central and peripheral tissues. Disruption of the network can lead to obesity and metabolic syndrome in mice (24).

**Table 5:** The top 5 literature in citations.

Rank	First author	Journal	Year	Citations	TLS
1	Turek, FW	SCIENCE	2005	609	14,636
2	Kohsak, A	CELL METAB	2007	425	10,523
3	Hatori, M	CELL METAB	2012	358	10,065
4	Scheer, FAJL	P NATL ACAD SCI USA	2009	307	8,426
5	Marcheva, B	NATURE	2010	281	8,114

The parameters selected for the co-cited reference analysis using CiteSpace were as follows: time slicing (January 1963–June 2024), years per slice (1), node type (cited reference), selection criteria (k = 15), and pruning (Pathfinder). As a result, a co-occurrence network with 1,490 nodes, 3,529 connections, and a density of 0.0032 was generated (Figure 7C). Afterward, we employed the Latent Semantic Indexing (LSI) approach with the “keywords option” for selection type to perform a cluster analysis of the cited references, which discovered 17 clusters. CiteSpace, by default, displays only the largest connected component (LCC) of the underlying network, meaning clusters that are not part of this LCC remain hidden. Therefore, Figure 7D shows only 10 clusters. To assess the quality of the clustering outcomes, we utilized the average profile (S value) and modularity (Q value) (38). According to the literature, clustering outcomes are deemed reliable when the Q value exceeds 0.5 and the S value exceeds 0.7 (39). In Figure 7D, the modularity Q is 0.8802 (> 0.5) and the S-value is 0.9378 (> 0.5), suggesting this clustering is well-structured and reasonable. These clusters can be grouped into three key areas of focus. Firstly, they encompass the pathogenesis of the circadian rhythm in obesity, with clusters such as #0 circadian rhythm, #4 endothelial nitric oxide synthase, #5 gene expression, #8 autonomic nervous system, and #17 eating disorders. Secondly, the treatment strategies for obesity are represented by clusters #1 restricted feeding and #2 intermittent fasting. Lastly, the clusters cover diseases associated with circadian rhythm that have been investigated in this discipline, including #3 anorexia nervosa, #6 metabolic syndrome, and #14 ambulatory blood pressure monitoring.

The evolution of these clusters is shown on a timeline in Figure 7E, where the earliest portions are identified as #3, #5, and #8. As shown on the timeline, recent lines of research in this field have emerged around topics such as circadian rhythm, intermittent fasting, endothelial nitric oxide synthase, ambulatory blood pressure monitoring. These fresh areas of investigation have gained attention in this discipline over the past several years.

Among the top 25 references with the strongest citation bursts (Figure 7F), the article by Wilkinson MJ et al. stands out with a burst intensity of 33.41, indicating its frequent citation in recent years. Their study found that after 10 h of time-restricted feeding (TRF) for 12 weeks, patients with metabolic syndrome getting standard medical care experienced improvements in cardiometabolic health. These improvements included weight loss, reduced atherosclerotic lipid levels, and lower blood pressure, suggesting that TRF could be a novel therapeutic option for individuals suffering from metabolic syndrome (40). Additionally, the article titled “Obesity and metabolic syndrome in circadian Clock mutant mice” published in 2005, had the strongest citation burst. This research proved mutations in the clock gene Clock can lead to significant alterations in fuel metabolism and metabolic characteristics associated with obesity, diabetes, and metabolic syndrome, indicating that the circadian clock gene network is crucial for maintaining energy balance in mammalian (24).

### 3.7 Keyword analysis

**Figure 8:**
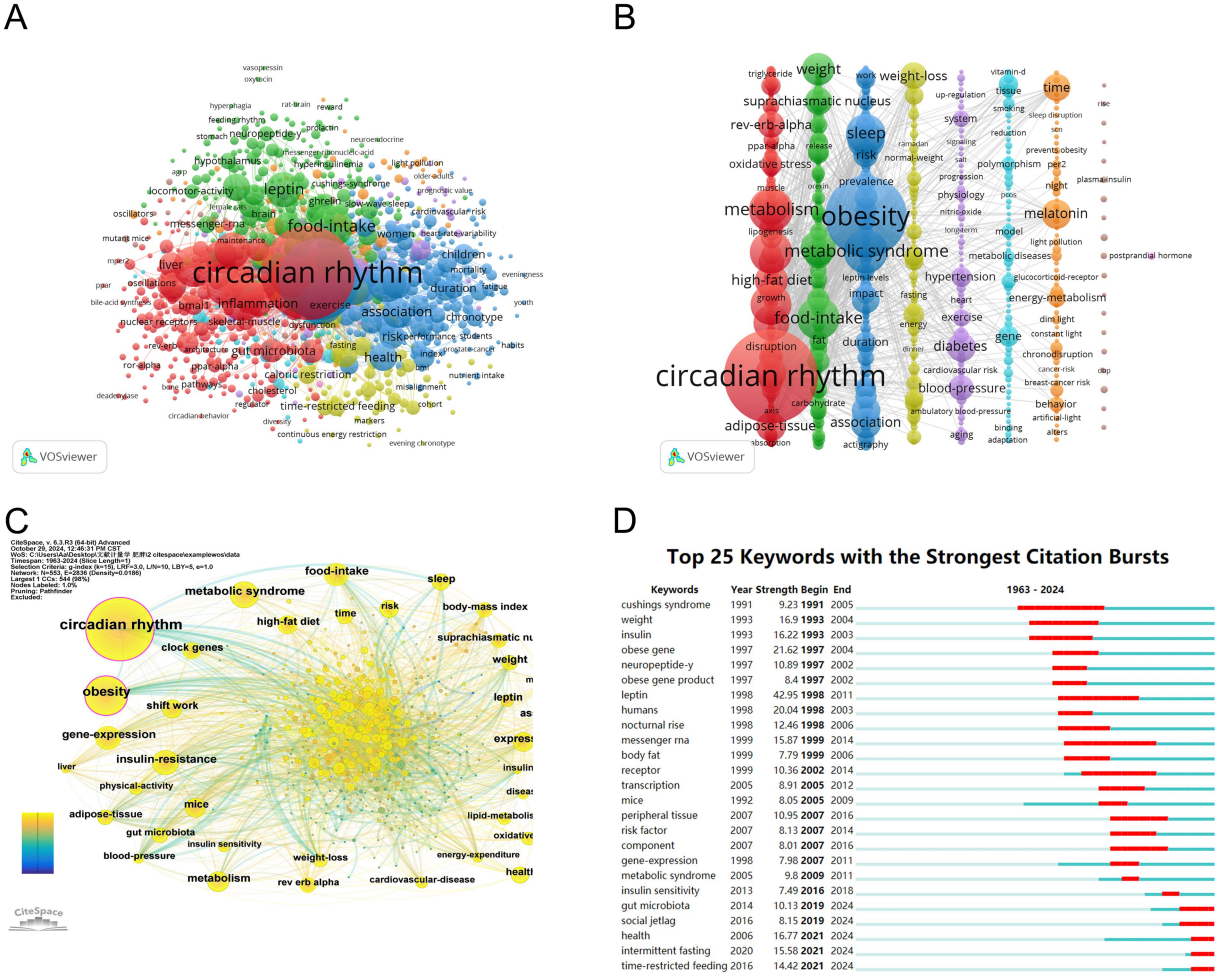
The mapping on keywords of the circadian rhythm research in obesity. **(A)** Keyword clusters in the studies of the circadian rhythm in obesity based on VOSviewer (divided into 9 clusters according to different colors). **(B)** Visualization of keyword clustering in the vertical form. **(C)** Visualization of keywords based on CiteSpace. **(D)** Top 25 keywords with the strongest citation bursts.

After combining terms with similar meanings, the top 10 keywords are summarized in Table 6. VOSviewer software was used to perform the co-occurrence network analysis of keywords. After being cleaned with a thesaurus, 907 out of 8,795 keywords met the minimal requirement of 5 occurrences. Further cluster analysis of the keywords (Figures 8A,B) obtained a total of 9 distinct colored clusters, each representing a different study path or research area.

**Table 6:** The top 10 keywords in terms of occurrences frequency.

Rank	Keywords	Occurrences	TLS
1	circadian rhythm	1900	16,337
2	obesity	1,434	12,716
3	metabolic syndrome	414	3,884
4	metabolism	408	3,726
5	food-intake	387	3,700
6	insulin-resistance	382	3,522
7	gene-expression	358	3,185
8	sleep	334	3,032
9	shift work	301	2,949
10	expression	282	2,385

The largest cluster is the cluster 1 (red), containing 232 keywords. This cluster primarily focuses on terms related to the circadian rhythm, metabolism, insulin-resistance, high-fat diet, rev-erb-alpha, oxidative stress, adipose-tissue, clock genes, gene-expression, inflammation, etc. Followed by the cluster 2 (green), with 204 keywords, including food-intake, weight, leptin, suprachiasmatic nucleus, insulin, glucose, fat, resistance, receptor, stress, etc. With 196 terms, cluster 3 (blue) includes obesity, metabolic syndrome, sleep, shift work, association, body-mass index, risk, physical-activity, prevalence, cardiovascular-disease and impact. Cluster 4 (yellow) has 77 keywords, mainly including weight-loss, caloric-intake, fasting, dinner, normal-weight, energy-expenditure, glucose-tolerance, caloric-intake, time-restricted feeding and intermittent fasting. 64 keywords make up cluster 5 (purple), which includes diabetes, blood-pressure, hypertension, physiology, system, aging, heart, exercise, cardiovascular risk and nitric-oxide. 61 keywords are found in Cluster 6 (light blue), including gene, model, polymorphism, tissue, metabolic diseases, susceptibility, epigenetics, DNA methylation, genetics, vitamin-d. Cluster 1 and 6 mainly reflect the regulatory mechanism of circadian rhythm leading to obesity, and Cluster 2 mainly reflects the influence of circadian rhythm on metabolism. The keywords in Cluster 3 and 5 are primarily related to lifestyle leading to obesity based on circadian rhythm changes, and the keywords in Cluster 4 primarily focuses on lifestyle management for the treatment of obesity.

The co-occurrence network of the keywords was also visualized using CiteSpace, yielding a map with 552 nodes, 2,848 connections, and 0.0187 density (Figure 8C). A keyword burst analysis identified the top 25 most cited keywords (Figure 8D). The five keywords with the highest burst strength were leptin (43.99), diurnal rhythm (31.78), obese gene (21.69), humans (20.13), and weight (17.44). The five keywords with the longest duration of burst were diurnal rhythm (1994–2010), messenger rna (1999–2014), receptor (2002–2016), cushings syndrome (1991–2005), leptin (1998–2011). From 1997 to 2007, the most frequently cited keywords included obese gene, neuropeptide-y, obese gene product, leptin, humans, nocturnal rise, obese, and body fat. In recent years, keywords such as gut microbiota, social jetlag, health, intermittent fasting, and TRF have emerged as focus points of research.

## 4 Discussion

Using VOSviewer, CiteSpace, and WPS, 2,870 publications from Web of Science were analyzed. Despite some fluctuations over the past 50 years, the results showed an overall rising trend in both the quantity of publications and annual co-citations. Notably, there has been a significant increase since 2013, reflecting growing interest among researchers in the connection between circadian rhythm and obesity.

The primary countries and regions contributing to research in circadian rhythm and obesity were the USA, Europe, and Asia. The USA held a leading position, with a significantly higher number of publications, citations, H-index, and Total Link Strength (TLS) compared to other countries, driving forward research in this field. Among the top 11 institutions with the highest number of publications, 7 institutions are located in the USA, while the remaining four institutions are in France or Spain, further illustrating the prominent role of the USA in this field. As shown in Table 3, the University of California System leads in the number of publications from the USA, conducting extensive research on the circadian rhythm in obesity. Recently, clinical trials demonstrated that 12 weeks of TRF within a self-selected 10 h eating window can significantly improve cardiac metabolic outcomes, including reductions in body weight, body mass index, waist circumference, glucose levels, and systolic blood pressure. They also emphasized the importance of studying the mechanisms of TRF, particularly its impact on circadian rhythm disorders (41). The significance of nodes in the network can be measured using centrality, a parameter where higher centrality means more frequent cooperation between countries or regions (42). In terms of centrality rankings, 7 institutions belong to the United States, the other institutions, respectively, locate in France, Spain, and Germany. China and Japan ranked second and third in terms of the quantity of papers published, but TLS was relatively low. Thus, it is recommended to enhance cooperation between countries and institutions to publish more influential articles and jointly advance the field of circadian rhythm research in obesity.

In terms of authorship, Garaulet, Marta ranked first among the top 10 authors with the most publications, followed by Oster, Henrik and Panda, Satchidananda. Garaulet, Marta reviewed scientific evidence showing the relationship between chronobiology and obesity, and concluded that chronobiology could be a valuable tool for the future treatment of obesity and metabolic syndrome through chronotherapy (43). Garaulet, Marta pointed out that the circadian rhythm health status of obese, overweight, and normal-weight children differs according to the assessment of a global circadian score (GCS), with children with obesity showing poorer circadian cycle health (44). Additionally, Turek FW was the most co-cited researcher, and his academic interest mainly focused on neurobiology, sleep, circadian rhythm, metabolism and gut microbiota. Through the observation of a genetic mouse model of obesity and diabetes (db/db mouse), he found that impaired leptin signaling has harmful effects on the regulation of sleep volume, sleep structure and time consolidation of wake states, leading to a decrease in circadian rhythms of activity (45).

The journal with the most publications in this field was Proceedings of the National Academy of Sciences of the United States of America, followed by Science. The American Journal of Physiology was the most frequently cited journal. The top ten journals with the most publications and the top thirteen journals with the highest centrality all belong to the JCR partitions of Q1 or Q2, indicating that researchers can prioritize these types of journals when publishing articles, as the publications involved in this analysis are of a dependable standard.

The co-cited references are considered foundational to research in a certain field, and the development of keywords can reflect the evolution of frontier knowledge. Both co-cited references and keywords play a crucial role in guiding future study directions in this field (46). In this study’s analysis, similar topics emerged in co-cited reference clustering and keyword clustering, suggesting that these topics represent popular research areas in this field. The gene expression of obesity was identified as an early hotspot in circadian rhythms and obesity studies, which may be related to the successful isolation of obese gene in rats and humans and the discovery of human obese gene structure (47, 48). In 1994, Joseph S Takahashi and his research group defined the CLOCK gene, which is essential for normal circadian rhythm behavior, proposing that the Clock gene product may be part of the circadian clock mechanism (49). Since then, scientists have gradually focused on the clock gene expression of obesity. At the molecular and cellular levels, the biological clock in adipose tissue is based on the interlocking transcription-translation feedback loop of clock genes and proteins. The expression patterns of clock genes such as BMAL1 and CLOCK show 24 h oscillations in adipose tissue (50). Driven by the CLOCK and brain and muscle ARNT-Like 1 gene (BMAL1) transcription factor dimers (51, 52), the molecular clock regulates the expression of thousands of genes, including period (PER) genes (PER1, PER2 and/or PER3) and cryptochrome (CRY) genes (CRY1 and CRY2) (53). When organisms are exposed to circadian rhythm disruptors, many biological systems and feedback loops, designed to predict and regulate circadian homeostasis, may become misaligned. Over time, this imbalance can negatively impact organs and tissues, disrupting catabolism and anabolic processes and leading to obesity (54). According to the timeline representing the evolution of clusters, endothelial nitric oxide synthase (eNOS) has been a research hotspot in recent years. Evidence suggests that eNOS signaling transduction is impaired in mice experiencing both obesity and constant darkness, although eNOS expression remains unchanged (55). However, the involvement of eNOS in circadian rhythm regulation remains unclear, and further research is needed to elucidate the role of eNOS in the fat clock (56).

With the research progress on circadian rhythm in obesity, several emerging fields have attracted increasing attention from researchers. One notable area is intermittent fasting. As obesity becomes an important public health issue, it has gained popularity in recent years as an approach to controlling obesity and weight loss (57). This may be attributed to the fact that intermittent fasting is an easier strategy for most people than increasing energy consumption through physical exercise (58). Intermittent fasting focuses on limiting eating to specific times within a day or week. This dietary intervention is similar to calorie restriction and typically includes two main types: alternative day fasting and TRF (59). A number of studies have shown that intermittent fasting can reduce body weight and fat by improving insulin resistance, lowering total cholesterol, low-density lipoprotein (LDL), triglycerides, and simultaneously elevating high-density lipoprotein (HDL) levels (60–62). TRF, a common form of intermittent fasting, involves limiting food intake to a specific time window—usually between 1 and 12 h—and avoiding calorie consumption for the rest of the day (63). Clinical trials have shown that TRF can change the expression of clock genes, restore the circadian rhythm of metabolites and glucose-regulated hormones, and establish the 24 h gene expression spectrum that regulates transcriptional control in human adipose tissue (64). Chambers et al. (65) critically evaluated the studies from PubMed and concluded that early circadian-aligned TRF diets are linked to increased diet-induced thermogenesis, improved blood glucose control, and weight loss. These findings suggest that circadian-aligned TRF diets may be an effective strategy for enhancing metabolic health and promoting weight loss in individuals who are overweight or obese.

Recently, the effects of TRF on the gut microbiome and body weight has gained attention. A study by Thaiss et al. revealed that the composition and function of the intestinal flora in laboratory mice fluctuated within a few hours, aligning with the 24 h rhythmic hypothesis of intestinal microbial function. It is proved that the rhythm of host biological clock-driven functions, such as the timing of food intake, is crucial to the daily oscillations that shape the composition of the microbiota (66). In multiple human and experimental models, melatonin has shown to improve gut microbiota imbalance related to metabolic status by regulating disrupted circadian rhythms, thus demonstrating beneficial effects on obesity (67, 68). Additionally, a preliminary randomized, double-blind, placebo-controlled study conducted on overweight and obese individuals with insulin resistance revealed that supplementation with live or pasteurized myxobacteria for 3 months increased insulin sensitivity and reduced insulinemia and total plasma cholesterol. Moreover, there was a slight reduction in fat mass and hip circumference compared to baseline (69). Therefore, in clinical practice, microbiome-targeted therapies could emerge as novel treatment options for obesity and metabolic syndrome (70).

The study of circadian rhythms and obesity may also have an important impact on clinical practice and public health policies. Through in-depth understanding of the relationship between circadian rhythm and obesity, a more personalized treatment plan can be developed from multiple perspectives such as diet, sleep, and gut microbiome to improve the therapeutic effect (44, 54, 71). Obesity is a major public health concern, and we believe it is important to strengthen interdisciplinary cooperation among medicine, nutrition, psychology, and other disciplines to inform the formulation of scientific public health policies. Shift work refers to the working mode of periodically changing working hours (72). It can disturb the circadian rhythm, leading shift workers to face a variety of health issues (73, 74). Numerous studies have shown that shift work may be associated with an increased risk of overweight and obesity (75–77). Therefore, shift schedules should be created according to recommended ergonomic standards to mitigate adverse health impacts by preventing or minimizing circadian rhythm disorders and insufficient sleep (78). Based on the research results of circadian rhythm and obesity, more scientific public health policies can be formulated, such as promoting healthy lifestyles, providing scientific dietary and exercise guidance, and raising public awareness of circadian rhythms. The WHO’s recent recommendations on obesity mention that in order to prevent childhood obesity, children should follow a healthy lifestyle and ensure sleep time and quality (79), indicating that the role of circadian rhythms in obesity is gradually being valued. Therefore, we recommend that more parts of the impact of circadian rhythms on obesity should be added to the guidelines issued by relevant international organizations in the future, helping people understand and maintain a healthy biological clock as a strategy for the prevention and treatment of obesity, thereby reducing its prevalence.

This study conducted a bibliometric analysis of the circadian rhythm in obesity, using Citespace and VOSviewer to comprehensively analyze and visualize the literature network. We summarized the current research landscape and identified key research forces in this field, and predicted future trends. However, due to the limitations of bibliometric tools, paper types and language, valuable newly published research results may have been overlooked, and important papers published in languages other than English may be missed. Our study was restricted to publications in the WoSCC database, as CiteSpace currently cannot analyze references from other databases. Future software improvements are expected to broaden the range of database selection for analysis. In addition, as the circadian rhythm in obesity is a developing research field, some recently published high-quality articles may be cited less frequently due to their short publication time, which may lead to differences between research results and actual situations.

## 5 Conclusion

In this study, we utilized the Web of Science database, along with CiteSpace and VOSviewer software, to conduct bibliometric and visual analysis of research on obesity and circadian rhythm. This approach provided a scientific and intuitive view of the overall research landscape in this field. According to bibliometric analysis, research on the circadian rhythm of obesity began in 1963, and the number of published articles has significantly increased over the past few decades. We conducted an in-depth analysis of publication data, covering various aspects such as the quantity of published articles, prominent countries and institutions, influential authors and co-cited authors, journals published, and cooperative networks. Furthermore, the study highlighted major research hotspots, development trends, and frontier topics related to obesity and circadian rhythm. As the understanding of the circadian rhythm mechanism deepens, the therapeutic targets related to circadian rhythms present promising prospects for obesity treatment. The investigation of obesity mechanisms and treatment methods that leverage circadian rhythm has emerged as a major research hotspot. Key areas of focus include circadian gene expression, intermittent fasting, restricted feeding, endothelial nitric oxide synthase, and gut microbiota. These fields highlight potential future research directions and offer valuable guidance for advancing studies in this field.

To sum up, our results may offer valuable insights for researchers to understand the current status and emerging trends in circadian rhythm research related to obesity. This analysis can serve as a reference and provide suggestions for guiding future research directions in this field.

## Data Availability

The original contributions presented in the study are included in the article/supplementary material, further inquiries can be directed to the corresponding authors.
